# A Report of Cauda Equina Syndrome Caused by Spinal Epidural Hematoma, a Complication of Deep Vein Thrombosis (DVT) Management

**DOI:** 10.7759/cureus.47969

**Published:** 2023-10-30

**Authors:** Tamam Mohamad, Satesh Kumar, Amir Kaki, Giustino Varrassi, Rick Markiewicz

**Affiliations:** 1 Cardiovascular Medicine, Wayne State University, Detroit, USA; 2 Medicine and Surgery, Shaheed Mohtarma Benazir Bhutto Medical College, Karachi, PAK; 3 Cardiology, Heart & Vascular Institute, Dearborn, USA; 4 Pain Medicine, Paolo Procacci Foundation, Rome, ITA; 5 Cardiology, Ascension St. John Hospital, Detroit, USA

**Keywords:** spinal epidural hematoma, dvt, ces, cauda equina syndrome, hematoma, epidural

## Abstract

This case report delves into the infrequent yet substantial occurrence of cauda equina syndrome (CES) arising from a spinal epidural hematoma (SEH), a potential complication during deep vein thrombosis (DVT) treatment. An 83-year-old female patient previously diagnosed with various medical conditions, including moderate spinal stenosis, chronic kidney disease, and chronic lower extremity stasis, was detected with notable iliofemoral DVT during an office-based venous study. The patient was urgently referred to the Emergency Department. Following hospitalization, her cardiologist diagnosed DVT, prompted by the patient's report of significant swelling and pain in the left lower limb over the past week. A venous ultrasound unveiled occlusive DVT originating from the common femoral vein, extending down to the infrapopliteal vessels, with a complete absence of venous Doppler signal. Employing the ClotTriever device (Terumo Corporation, Shibuya City, Tokyo, Japan), a mechanical aspiration thrombectomy procedure, effectively resolved DVT. However, CES was diagnosed in the patient on a subsequent day due to the emergence of SEH. This case underscores the intricate balance required when managing DVT, involving anticoagulation or alternate therapies while acknowledging the potential risk of hemorrhagic complications leading to epidural hematoma and consequent CES. It is crucial for clinicians managing DVT and employing therapeutic strategies to be aware of this infrequent yet pivotal complication. This report highlights the significance of prompt identification and intervention in such cases, emphasizing the need for vigilance and understanding of potential complications during DVT treatment.

## Introduction

Spinal epidural hematoma (SEH) is a rare yet potentially severe ailment with the capacity to induce acute cauda equina syndrome (CES), characterized by substantial morbidity and neurologic impairments [1]. SEH is typified by the accumulation of blood within the spinal epidural space, which can lead to the compression of the spinal cord and/or cauda equina nerves [2]. While spontaneous SEH is uncommon, it is typically associated with trauma, anticoagulant therapy, vascular anomalies, spinal epidural procedures, or spinal surgery [3].

The clinical presentation of SEH hinges on the hematoma's location and size within the spinal canal. Manifestations often encompass severe pain, neurologic deficits, and the development of CES. The latter syndrome is recognized by sensorimotor impairments, gastrointestinal and urinary dysfunction, and potential paralysis of affected limbs [3]. Timely and precise assessment of patients' clinical status, coupled with the employment of diagnostic imaging modalities, is of paramount significance for the timely identification of potential health concerns, thereby facilitating the implementation of appropriate therapeutic strategies. Surgical evacuation of the hematoma is frequently necessary upon confirmed diagnosis. The prognosis is influenced by multiple variables including the speed of symptom onset, the interval between diagnosis and surgery, the degree of spinal involvement, and the level of compression [4].

This case study specifically examines the clinical presentation and diagnostic challenges encountered subsequent to deep vein thrombosis (DVT) treatment. The primary objective of this report is to heighten awareness and comprehension of this intricate condition within the medical community. The prompt identification of medical conditions holds exceptional importance in mitigating severe complications and optimizing patient prognosis. This case report is anticipated to serve as a valuable educational resource for clinicians in the accurate identification and management of epidural hematoma following DVT treatment.

## Case presentation

An 83-year-old female with a medical history encompassing moderate spinal stenosis, chronic kidney disease, and chronic lower extremity stasis presented with a unique constellation of medical challenges. The patient was referred to the Emergency Department following a venous study conducted within the clinic, which unveiled a significant iliofemoral DVT. Notably, her medical journey had previously included admission to another healthcare facility, where she was diagnosed with cellulitis and subsequently discharged to her residence. Of paramount concern was DVT within the common femoral vein, extending to the infrapopliteal vessels, as illuminated by venous ultrasound. The patient's complaint of substantial swelling and persistent pain in the left lower extremity over the preceding week further underscored the severity of her condition. These clinical indicators prompted a rigorous course of action to tackle the intricate pathology.

**Figure 1:**
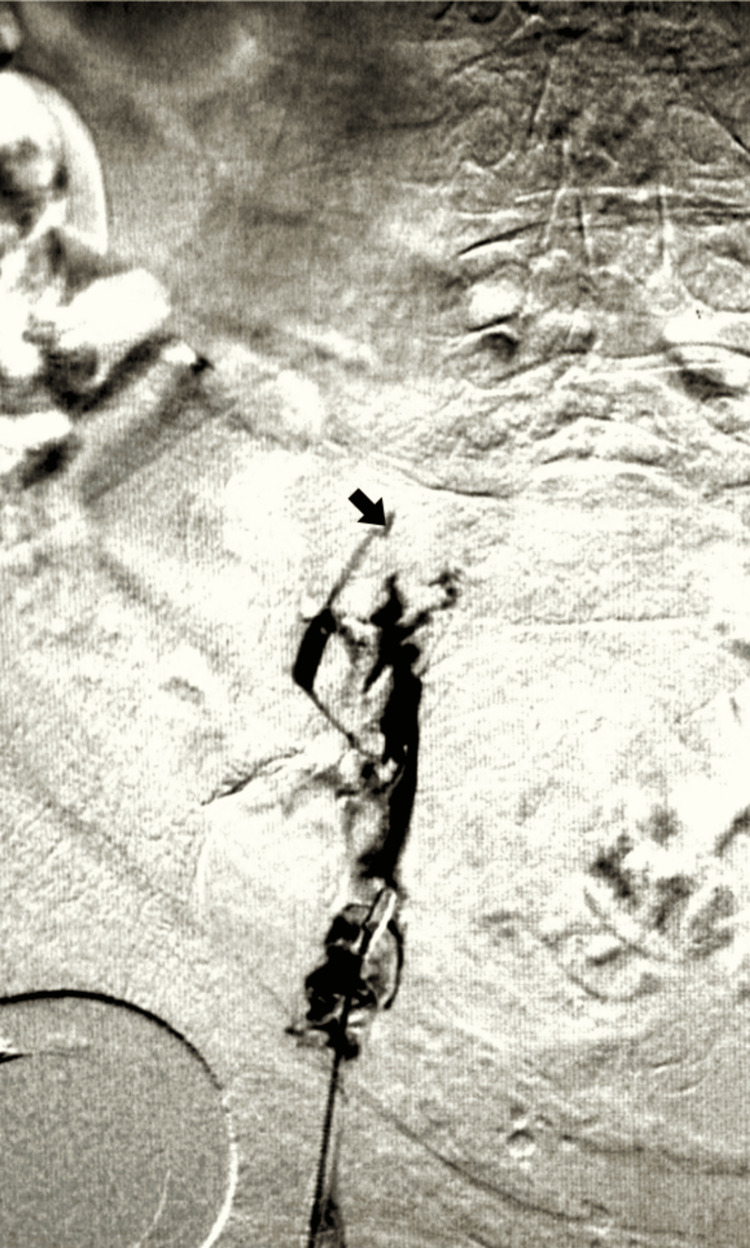
Prone position venography showing occlusive deep vein thrombosis (arrow) in the femoral vein, accompanied by chronic iliac vein occlusion.

The patient's case progressed to the catheterization lab, where a specialized intervention was orchestrated. Employing the technique of left lower extremity mechanical aspiration thrombectomy, the procedure unveiled an occlusive DVT within the femoral vein, coupled with chronic occlusion of the iliac vein, as evidenced by prone position venography (Figure 1).

**Figure 2:**
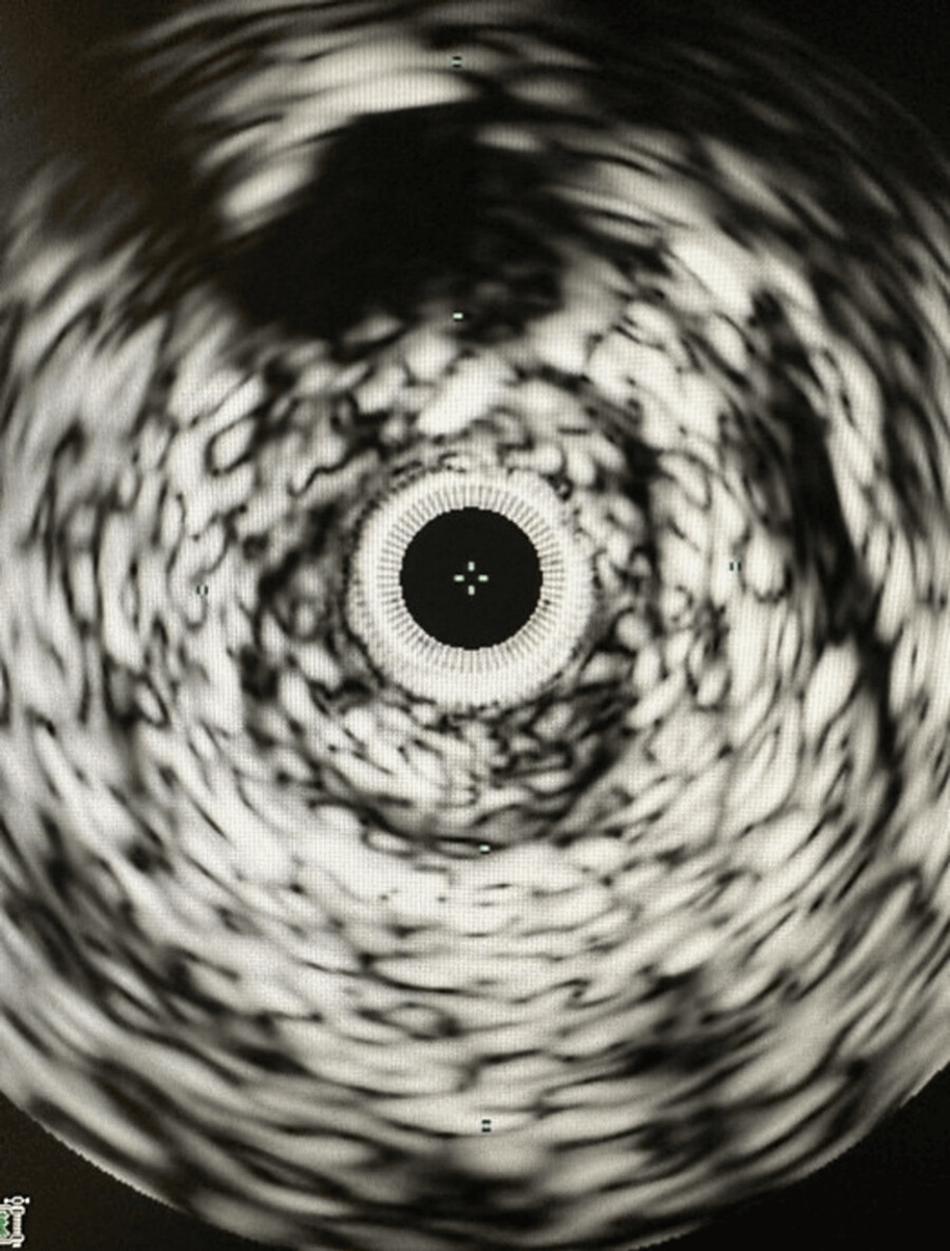
Intravascular ultrasound examination of the femoral vein, illustrating the presence of both acute and chronic thrombi.

Further insight into the nature of the thrombus was gleaned through an intravascular ultrasound (IVUS) examination of the femoral vein, delineating the presence of both acute and chronic thrombi (Figure 2).

However, the road to resolution encountered unforeseen hurdles. An endeavor to address the iliac vein occlusion via a 0.035 GLIDEWIRE ADVANTAGE® peripheral guidewire (Terumo Corporation, Shibuya City, Tokyo, Japan) and NaviCross™ support catheter (Terumo Corporation) through popliteal access was frustrated as success eluded the attempts. The clinical team, responding with adaptability, initiated a 48-hour intravenous heparin regimen to pave the way for a subsequent venogram and thrombectomy procedure. During this interim period, the patient's need for intravenous narcotics underscored the profound discomfort she endured. Notably, the presence of weeping wounds on the left leg, suggestive of venous phlegmasia, added complexity to her clinical trajectory.

**Figure 3:**
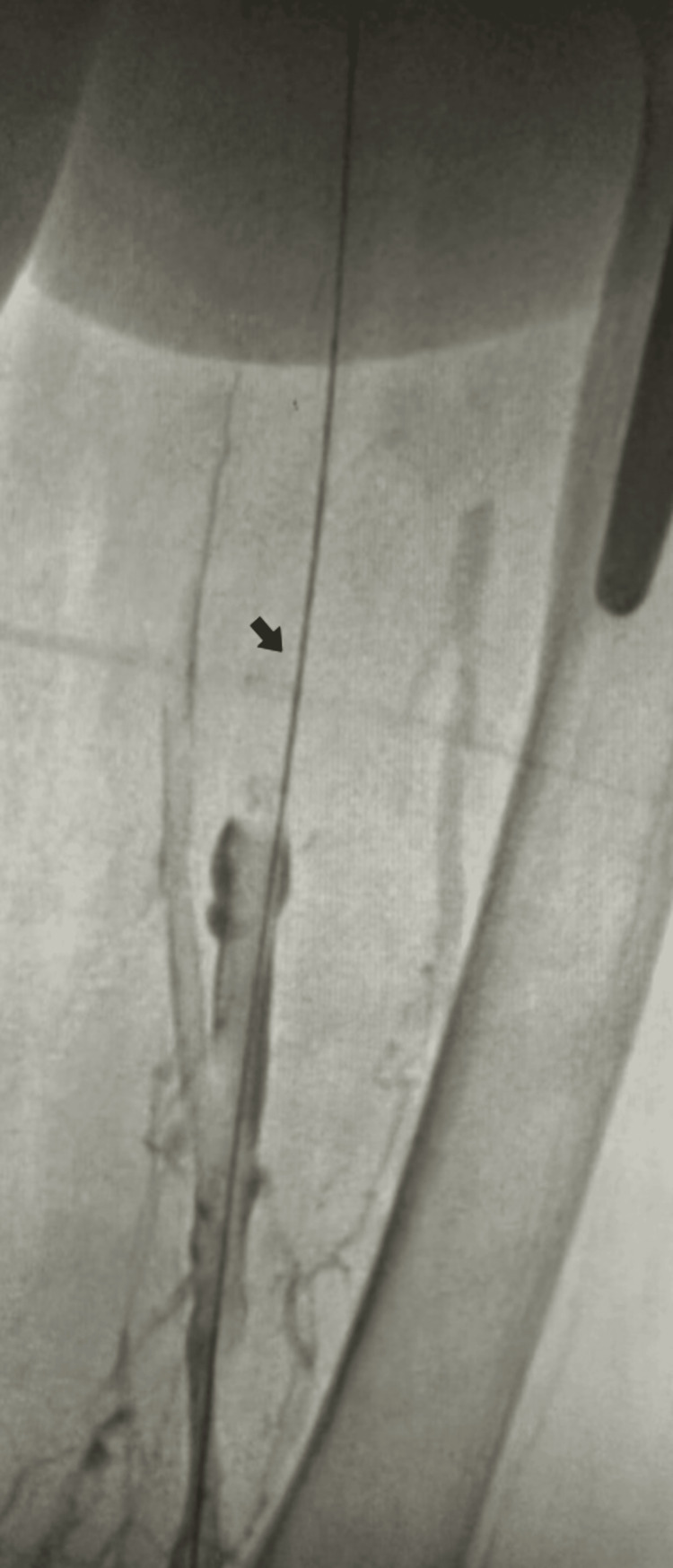
Subsequent angiographic evaluations showing persistent inflow occlusion. The arrow points to the enduring occlusion on angiogram, indicating no improvement in inflow.

Subsequent evaluations via angiogram, undertaken from a consistent access point, unfortunately, yielded no improvement in inflow to the chronic occlusion, as illustrated in Figure 3.

**Figure 4:**
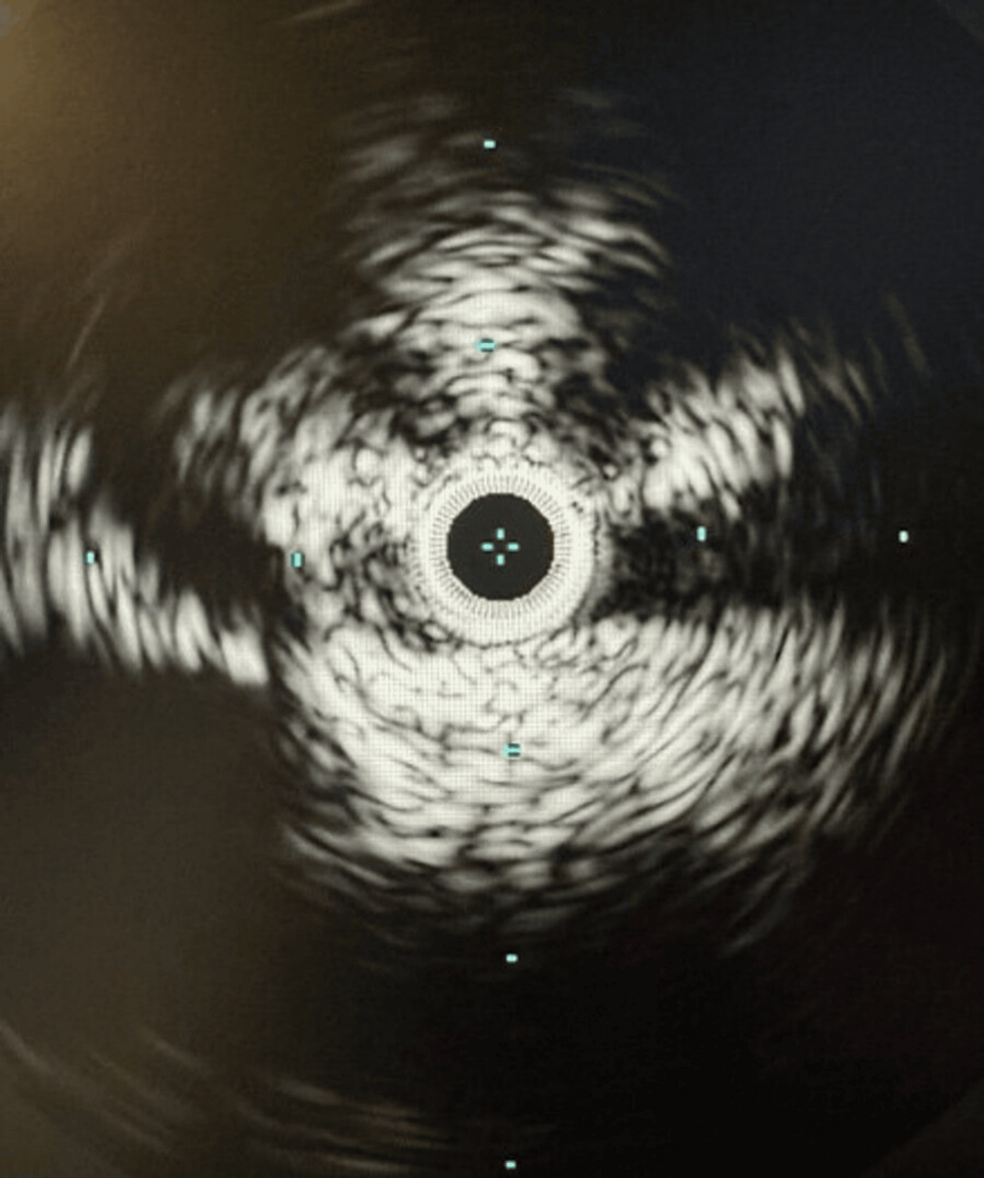
Intravascular ultrasound imaging illustrating constriction within the left common iliac vein.

As a strategic maneuver, the popliteal sheath dimensions were augmented, with the introduction of a TriForce® Peripheral Crossing Set (Cook Group Incorporated, Indiana, United States) further enabled by a combination of angled sheaths and catheters. This meticulous orchestration facilitated the precise navigation of the common femoral artery, culminating in the successful traversal of the inferior vena cava (IVC) by inserting a straight stiff glide wire. This complex procedural intricacy was underscored by IVUS visualization, which revealed significant compression within the left common iliac vein (Figure 4).

**Figure 5:**
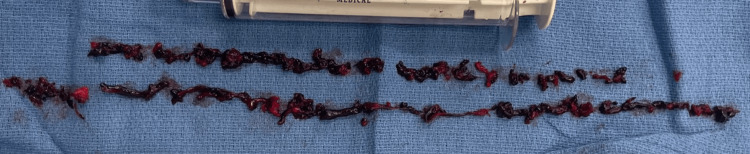
Utilization of the ClotTriever device* for mechanical aspiration thrombectomy, resulting in effective extraction of a significant quantity of subacute thrombus. *Terumo Corporation, Shibuya City, Tokyo, Japan

A preliminary (IVUS percutaneous transluminal angioplasty (PTA) procedure primed the terrain for the subsequent placement of an IVUS catheter. The subsequent phase of the case encompassed a mechanical aspiration thrombectomy procedure using the ClotTriever device (Terumo Corporation). This intervention removed a notable volume of subacute thrombus (Figure 5).

**Figure 6:**
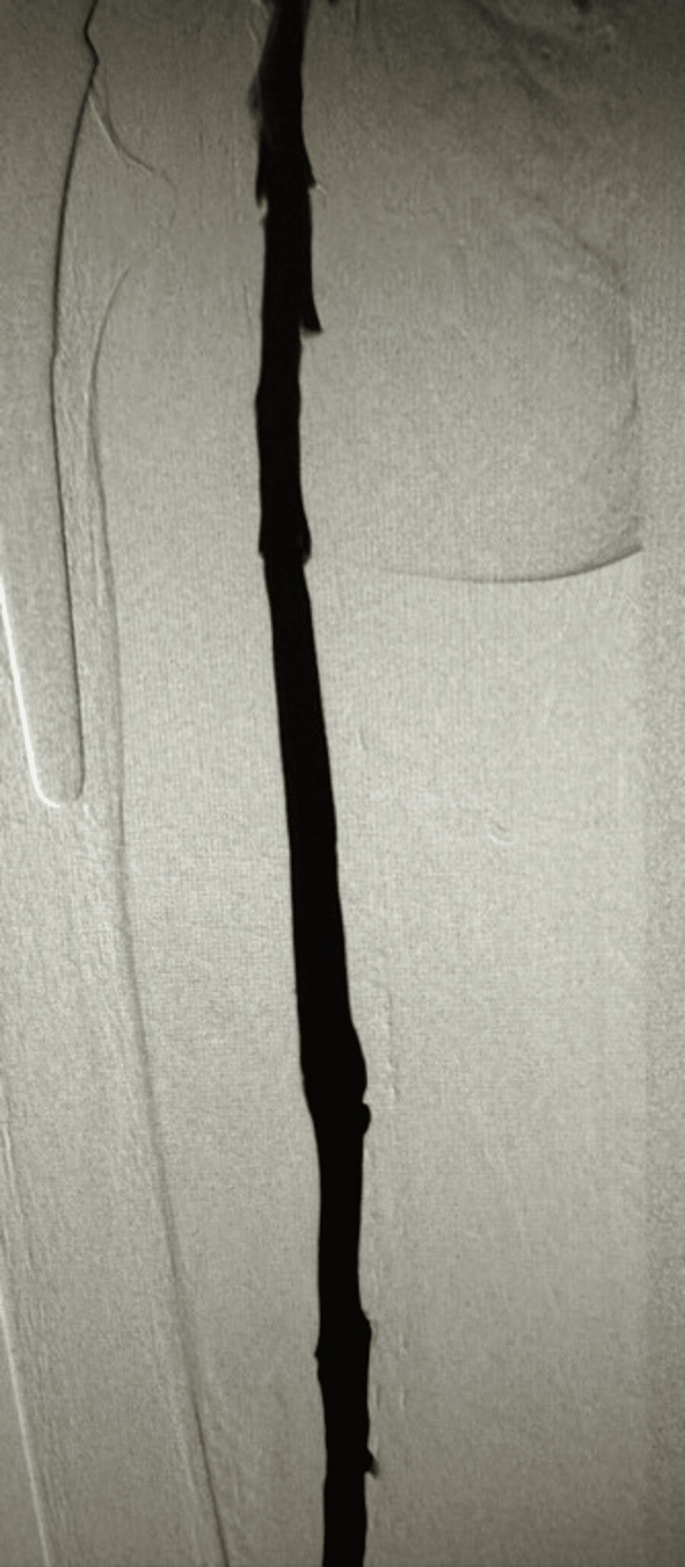
The epidural plexus assuming a supplementary function as an alternate route for venous drainage.

**Figure 7:**
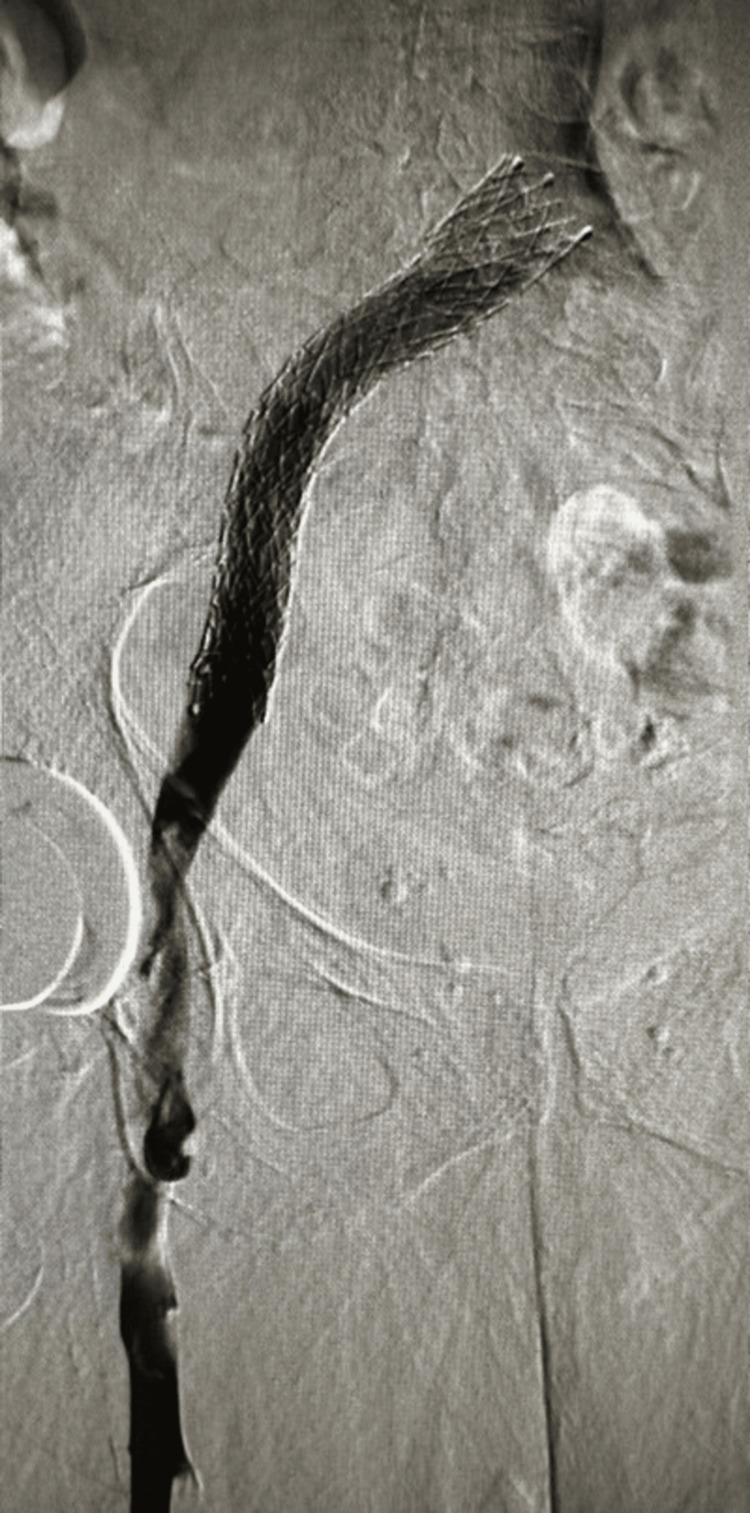
Venography depicting a satisfying resolution.

**Figure 8:**
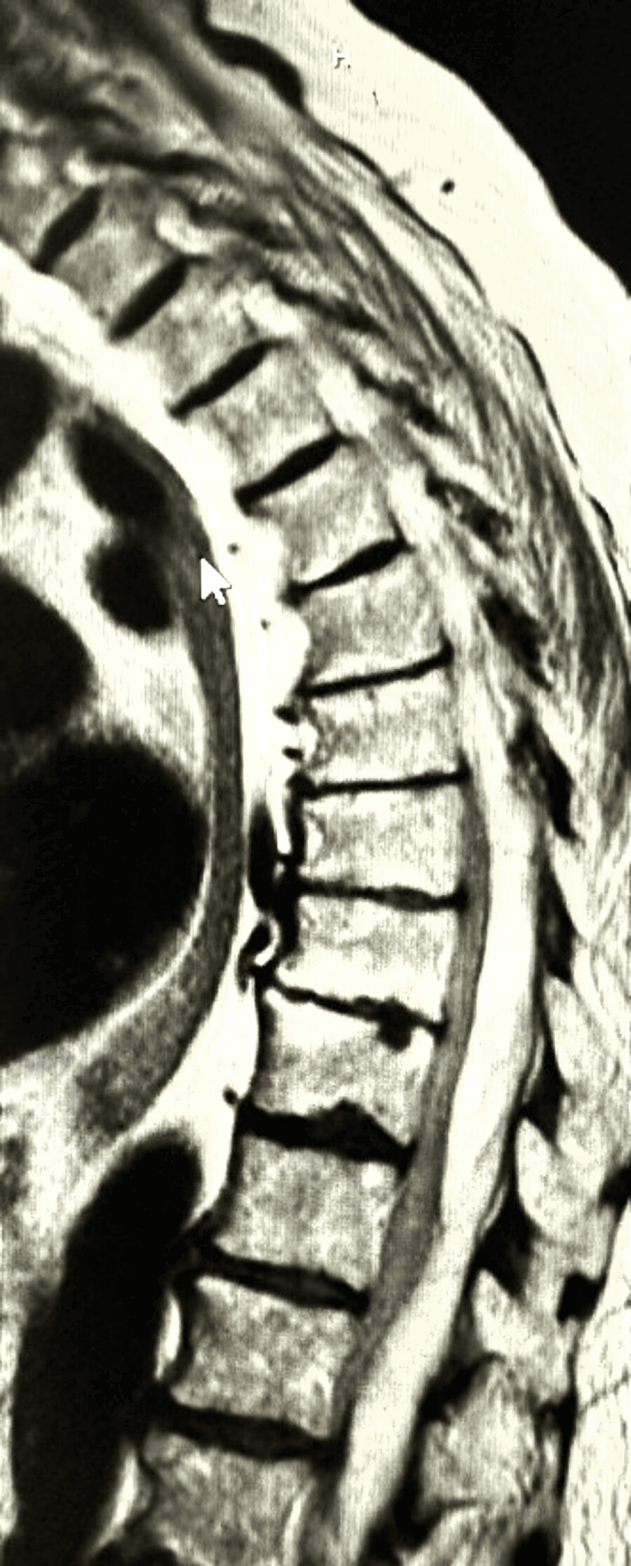
MRI revealing an epidural hematoma (arrow) within the posterior spinal canal extending from T6 to the lower sacrum, accompanied by fluid accumulation at T9 and T11 levels.

In a concerted effort, an extensive balloon PTA was directed toward the left iliac vein. Subsequently, placing a 16 by 150 Abre stent (Medtronic plc, Dublin, Ireland) further augmented the vascular architecture. The outcome of this intricate intervention was vividly portrayed through repeated venography, highlighting a gratifying resolution (Figures 6, 7). Figure 8 illustrates the presence of an epidural hematoma located in the posterior spinal canal spanning from the T6 to the lower sacral region, along with the observation of fluid accumulation at the T9 and T11 levels.

The patient's therapeutic journey embraced the administration of Plavix and intravenous heparin. At the same time, the prompt alleviation of leg congestion validated the efficacy of the therapeutic regimen. However, a subsequent phase brought forth unexpected clinical developments. The patient was administered a significant quantity of intravenous narcotics in response to the pain accompanying the interventions. The patient independently ventured to the bathroom overnight, only to awaken to a distressing reality. Paralysis and loss of sensation in her lower extremities greeted the morning's light. With the immediate discontinuation of heparin and Plavix, the clinical team embarked on a diagnostic trajectory encompassing an MRI scan and consultation with a neurosurgeon.

These interventions unveiled a posterior SEH, spanning from T6 to the lower sacrum, resulting in compression upon the spinal cord and cauda equina. The presence of fluid accumulation at T9 and T11 added a layer of complexity, exacerbating contraction against the vertebral bodies, emanating from T7 and spanning the length of the cord. Notably, bilateral defects in the pars of the L5 vertebra, coupled with moderate bilateral stenosis of the neural foraminal at the L5 to S1 level, further compounded the intricate landscape. Despite an emergent laminectomy procedure, two months elapsed without observed neurological improvement. The patient's case, replete with twists and challenges, offers a profound testament to the intricate interplay of medical conditions and the critical need for meticulous clinical management.

## Discussion

In the context of the intricate dynamics of the epidural venous complex, unraveling its complexities poses a challenge. The susceptibility of the epidural venous plexus to injury arises from a spectrum of underlying factors, spanning from occlusion of the vertebral artery and pelvic vein congestion to lumbar spinal canal stenosis [5]. This plexus, an intricate network of veins residing within the spine's epidural space, plays a crucial role in the drainage of venous blood and is implicated in a myriad of spinal and pelvic disorders. The engorgement of these veins can subsequently contribute to the development of radiculopathy and an array of neurological symptoms [6]. A comprehensive comprehension of these venous plexuses' intricate anatomy and potential complications is imperative, as it underpins accurate diagnosis and targeted treatment approaches.

**Figure 9:**
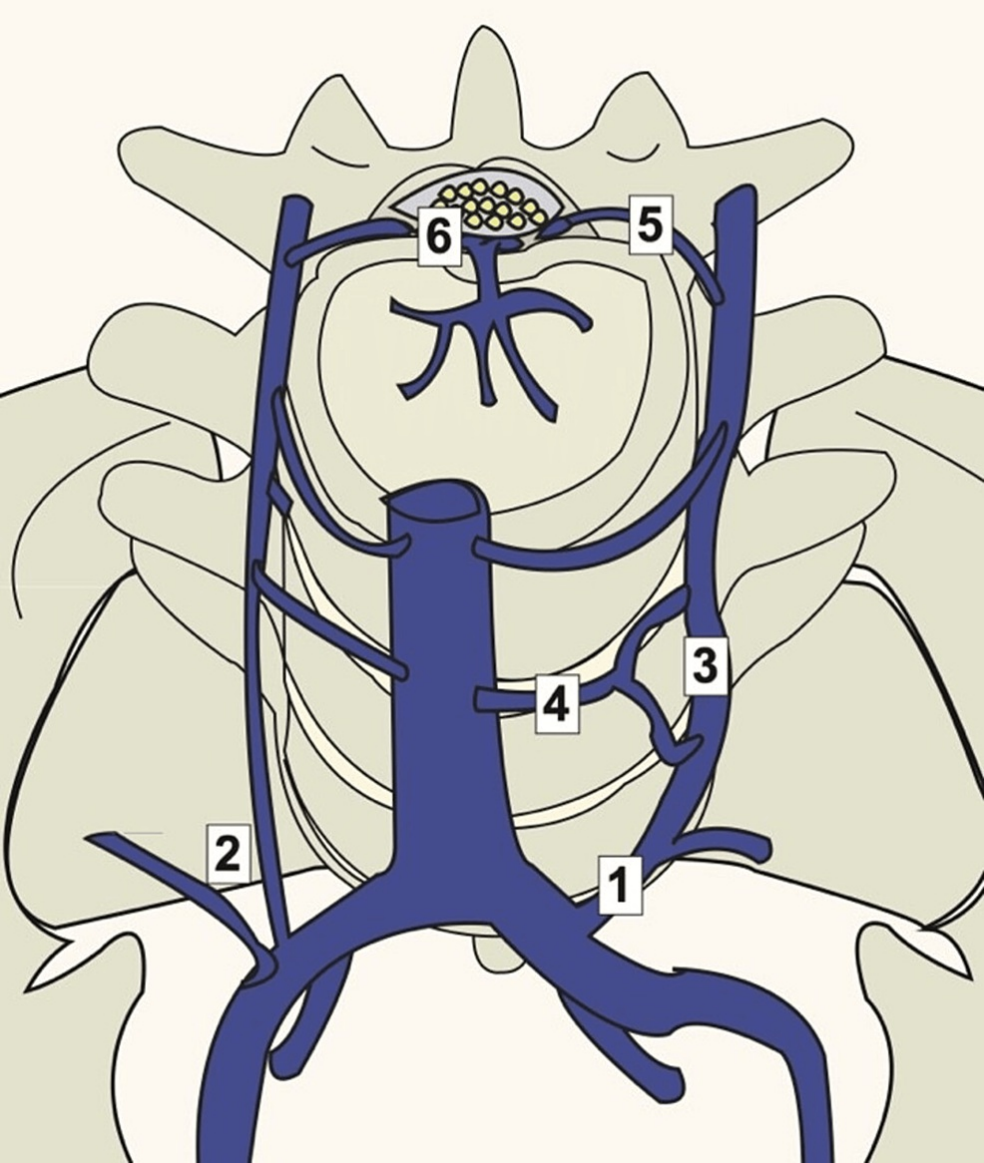
Illustration depicting the lumbar venous anatomy. The ilio-lumbar and 19 ascending lumbar veins can exhibit a shared origin (1) or a dual origin (2). The ascending lumbar vein (3) connects with the inferior vena cava (4) through the segmental lumbar veins. Foraminal Veins (5) link with the epidural venous plexus of the spinal canal (6). Image Source: Zaldivar-Jolissaint et al., 2020 [8]; Used with permission

MRI is a pivotal tool for visualizing the pathology within the spinal epidural venous plexus, affording the ability to pinpoint engorged epidural veins and their consequent impact on neighboring structures. When addressing the challenging scenario of iliocaval thrombosis and chronic occlusions, the epidural plexus takes on an auxiliary role as an alternative pathway for venous drainage, as shown in Figure 9 [7].

However, this plexus, vulnerable to damage by wires and catheters during the process of reconstruction, may encounter swelling and subsequent complications. The configuration of the ilio-lumbar and ascending lumbar veins showcases diversity, manifesting as either a common trunk origin or a double origin. The ascending lumbar vein establishes a connection to the IVC through the segmental lumbar veins, while the foraminal Veins forge a link with the epidural venous plexus of the spinal canal [9]. The emergence of epidural hematomas, characterized by the accumulation of blood between the dura mater and the skull, introduces a potentially life-threatening dimension [9,10]. Notably, the procedure known as iliocaval repair, commonly employed to address the intricacies of chronic DVT, holds the latent capacity to disrupt the epidural venous plexus, thereby forming an epidural hematoma.

Iliocaval repair, a therapeutic recourse designed to counter persistent obstructions within the iliac veins and the IVC [11], traverses precarious territory. The epidural venous plexus, responsible for the drainage of veins adjacent to the spinal column, can sustain inadvertent damage during the execution of the iliocaval repair procedure, forming an epidural hematoma. Applying undue pressure on neuronal structures within the spinal canal amplifies the potential risk [10]. Within this complex interplay, the current epidemiology and management of this relatively uncommon scenario remain obscured, awaiting further investigation. It is incumbent upon healthcare practitioners to consider the plausibility of epidural venous plexus injury in the context of iliocaval repair. Thus, the importance of cautious deliberation to safeguard the epidural venous plexus looms large, minimizing the inherent risks associated with the emergence of epidural hematoma and its cascading complications, including the ominous CES. The etiology of CES often converges around IVC thrombosis, which has been well-documented in the medical literature, possibly attributed to hydrostatic forces within the epidural space [8]. However, the intricacies of CES extend beyond the purview of this paper.

Amid the challenges posed by intricate clinical environments, it remains incumbent upon surgeons, anesthesiologists, and radiologists to forge a collaborative alliance that ensures patient safety and optimal outcomes. The case illustrated here stands as a testament to successful procedural execution, and the prospect of encountering such complications undoubtedly instills distress in both the medical practitioner and the patient. An unwavering commitment to avert such outcomes is paramount, with interventions such as cone beam CT and frequent lateral projections serving as proactive measures to forestall potential harm to the plexus. Bolstered by an exhaustive understanding of the anatomy and pathophysiology of the epidural space, the healthcare community stands fortified in its pursuit of safe and efficacious interventions.

## Conclusions

Managing DVT in this case exhibited commendable success, underscoring the efficacy of the chosen therapeutic approach. It is important to acknowledge that the emergence of intricate complications, notably CES stemming from epidural hematoma, is exceedingly infrequent. However, as healthcare practitioners navigate the landscape of DVT treatment, it becomes imperative to integrate a heightened awareness of this rare complication into the therapeutic decision-making process. Vigilant consideration and incorporation of appropriate management strategies aimed at preemptively addressing this potential complication will help mitigate its occurrence and uphold patient well-being. This nuanced approach aligns seamlessly with the commitment to delivering optimal patient care within complex medical interventions.
